# Integrated Transcriptome and Metabolome Analyses Reveal the Roles of MADS-Box Genes in Regulating Flower Development and Metabolite Accumulation in *Osmanthus fragran*

**DOI:** 10.3390/cimb47100819

**Published:** 2025-10-03

**Authors:** Qian Zhang, Jie Yang, Xiangling Zeng, Hongguo Chen, Yingting Zhang, Guifu Zhang, Zeqing Li, Xuan Cai, Jingjing Zou

**Affiliations:** 1 National Forestry and Grassland Administration Engineering Research Center for *Osmanthus fragrans*, Hubei University of Science and Technology, Xianning 437100, China; qianzhang@hbust.edu.cn (Q.Z.);; 2 Osmanthus Innovation Center of National Engineering Research Center for Floriculture, Hubei University of Science and Technology, Xianning 437100, China

**Keywords:** multi-omics integration, *MADS-box* gene family evolution, cis-regulatory element analysis, floral organ morphogenesis, tissue-specific gene expression, gene duplication

## Abstract

The MADS-box transcription factors play essential roles in various processes of plant growth and development. Here, we identified 107 *MADS-box* genes in *Osmanthus fragrans* Lour. genome (*OfMADS*), encoding proteins ranging from 61 to 608 amino acids. Phylogenetic analysis classified these genes into five subfamilies: MIKC*, *MIKCC*, *Mα*, *Mβ*, and *Mγ*, with conserved motif architectures within subfamilies. Tandem and whole-genome duplications were identified as key drivers of *OfMADS* expansion. Cis-regulatory element analysis revealed enrichment for hormone response and developmental regulatory motifs, implicating roles in growth and flowering processes. Transcriptome dynamics across six floral developmental stages (bolting to petal shedding) uncovered 78 differentially expressed *OfMADS* genes, including 16 exhibiting flower-specific expressions. Integrated metabolome profiling demonstrated robust correlations between critical *OfMADS* regulators and scent metabolites. This nexus suggests a potential role of these *OfMADS* in regulating specialized metabolite biosynthesis pathways. Our multi-omics study provides insights into the regulatory hierarchy of *OfMADS* in coordinating floral morphogenesis and the accumulation of economically significant metabolites in *O. fragrans*. These findings establish a foundation for subsequent functional validation and molecular breeding of horticultural traits.

## 1. Introduction

The MADS (Mcm1/Agamous/Deficiens/Srf)-box transcription factor gene family defined by the conserved 58–60 amino acids MADS domain (derived from initials of four transcription factors that were first discovered of this family: *MINICHROMOSOME MAINTENANCE 1* (*MCM1*), *AGAMOUS* (*AG*), *DEFICIENS* (*DEF*), and *SERUM RESPONSE FACTOR* (*SRF*)), represents a pivotal class of transcription factors with ubiquitous distribution across eukaryotes, particularly in the processes of floral organ differentiation, regulation of flowering time, and fruit development and ripening in angiosperms [1,2,3,4,5,6,7,8,9,10,11].

Phylogenetic analyses categorize *MADS-box* genes into two primary types: type I (subdivided into Mα, Mβ, and Mγ subgroups) and type II (referred to as MIKC-type genes) [5]. *MIKC* genes are further categorized into *MIKCC*-type and MIKC* type genes, with the former being the most extensively studied due to their essential roles in plant growth and development [5,12,13,14,15,16,17,18]. Structurally, *MIKCC*-type genes form the backbone of the ABCDE model of floral organ identity, where combinatorial interactions of A-, B-, C-, D-, and E-class genes specify the development of sepals (A + E), petals (A + B + E), stamens (B + C + E), carpels (C + E), and ovules (C + D + E) [19,20,21,22,23,24,25,26,27]. Beyond floral organogenesis, *MIKCC*-type genes also regulate flowering time and root development, underscoring their functional diversity [20,28,29,30,31,32,33]. In *Arabidopsis thaliana*, this model includes several well-characterized genes: *APETALA1* (*AP1*), an A-class gene, is expressed in sepals and petals [29]; *PISTILLATA* (*PI*) and *APETALA3* (*AP3*), classified as B-class genes, are expressed in petals and stamens [30]; the C-class gene *AGAMOUS* (*AG*) is expressed in stamens and carpels [31]; the D-class gene *AGAMOUS-LIKE 11* (*AGL11*), also known as *SEEDSTICK* (*STK*), is expressed in ovules [32]; and E-class genes, including *SEPALLATA1* (*SEP1*), *SEP2*, *SEP3*, and *SEP4*, are expressed across all four floral whorls [32,33].

*O. fragrans*, a renowned ornamental and aromatic woody plant, holds significant horticultural value due to its fragrant flowers and diverse floral traits [34,35,36]. In recent years, studies on *O. fragrans MADS-box* genes have uncovered species-specific regulatory mechanisms that extend beyond conserved models [37,38,39,40]. For example, Li et al. (2023) identified a novel flowering regulation strategy involving transcript isoform competition: key flowering genes *OfAPETALA1* (*OfAP1*) and *OfTERMINAL FLOWER 1* (*OfTFL1*) produce alternative isoforms that compete for binding partners to either promote or delay flowering [36]. Wang et al. (2022, 2024) demonstrated that *OfBFT* (*BROTHER OF FT AND TFL1*)*-a* and *OfBFT-b* are essential for flower formation in *O. fragrans* [37,38]. Additionally, Zeng et al. (2021) cloned and functionally validated *PISTILLATA/GLOBOSA-like1* (*OfGLO1*) could reduce stamen length, and lower seed set, confirming its conserved role in specifying stamen and petal identities [39].

The identification and characterization of *MADS-box* genes are of great significance for investigating flowering time regulation and flower organ development in plant species; yet, current research on *MADS-box* genes in *O. fragrans* is largely confined to the functional analysis of a limited number of key members [40,41,42,43]. We conducted a comprehensive genome-wide analysis of the *MADS-box* gene family in *O. fragrans* ‘Liuye Jingui’, a widely cultivated genotype valued for its intense fragrance and ornamental flowers [35]. The findings will advance our understanding of *MADS-box* gene evolution and function in woody plants and lay a critical foundation for molecular breeding of *O. fragrans* traits with horticultural and economic significance.

## 2. Materials and Methods

### 2.1. Data Sources and Identification of MADS-Box Genes in Osmanthus fragrans

The genome data of *Osmanthus fragrans* were obtained from the *Osmanthus fragrans* database (https://yanglab.hzau.edu.cn/OfIR/download/, accessed on 19 May 2025) [35]. Protein sequence of *Arabidopsis thaliana* (L.) Heynh., *Oryza sativa* L., *Malus domestica* Borkh., and *Vitis vinifera* L. were downloaded from TAIR, RGAP and Phytozome, respectively [44,45,46]. The hidden Markov Model (HMM) file of PF00319 was acquired from InterPro database [47]. Subsequently, HMMER 3.0 was used to identify potential MADS-domain proteins in above file species (E-value ≤ 1 × 10^−5^, similarity > 50%). To enhance search proteins, a novel HMM was constructed using the hmmbuild program [48]. Concurrently, BLASTP was conducted using AtMADS and OsMADS protein sequences as references (E-value ≤ 1 × 10^−5^, similarity > 50%) [49]. Candidate MADS proteins were further validated for the presence of conserved domains were using Pfam-scan (v30.0) software. Finally, the R package SeqFinder (v0.1) (https://github.com/yueliu1115/seqfinder, accessed on 19 May 2025) was used to select and retain the longest transcript isoform.

### 2.2. Phylogenetic Analysis of OfMADS Genes

Muscle (v3.8.1551) software was used to perform multiple sequence alignment of the OfMADS, AtMADS, OsMADS, MdMADS, and VvMADS proteins [50]. Subsequently, we constructed a phylogenetic tree using IQ-TREE (v2.0.3) employing the maximum likelihood (ML) method with bootstrap values derived from 1000 replicates, and visualized the tree using the R package ggtree (v3.10.0) [51,52].

### 2.3. Domain, Gene Structures, Conserved Motifs Analysis of OfMADS Genes

Domain and positional information were determined utilizing the Pfam-Scan software with Pfam database [53]. Conserved motifs were analyzed by MEME (v5.1.1) software with the maximum number of 15 and optimum widths of 30–100 amino acids, with subsequent extraction performed by Python (v3.13.3) scripts [54]. The exon and intron locations of *OfMADS* genes were derived from GFF3 annotation files. Visualization of domains, conserved motifs and gene structures were accomplished using the R package ggtree (v3.10.0) and gggenes (v0.5.2) (https://github.com/wilkox/gggenes, accessed on 19 May 2025).

### 2.4. Analysis of Chromosome Distribution, Gene Duplication Events, and Selection Pressure

The chromosomal distribution of *OfMADS* genes was determined using the GFF3 annotation file. Gene duplication and collinearity analyses were conducted with the MCScanX (v.transposed) software, which identified the duplication types, including tandem duplications (TD) and whole genome duplications (WGD) [55]. For the analysis and visualization of inter-species collinearity, the JCVI (v1.5.4) software was employed. The alignment of protein sequences and coding sequences (CDS) of *MADS* genes with gene duplication wes performed using ClustalW (v2.1) software [56]. Furthermore, the KaKs_Calculator (v2.0) software was utilized to compute the synonymous substitution rate (Ks), nonsynonymous substitution rate (Ka), and the evolutionary ratio (Ka/Ks) between duplicate gene pairs of *MADS-box* genes [57].

### 2.5. Analysis of Cis-Elements in the Promoter of OfMADS Genes

The 2 kb promoter region sequences located upstream of the *OfMADS* gene were extracted using a Python program and subsequently submitted to the PlantCare database (https://bioinformatics.psb.ugent.be/webtools/plantcare/html/, accessed on 19 May 2025) for prediction of cis-regulatory elements [58]. The results were comprehensively visualized using the R packages ggtree and gggenes (https://github.com/wilkox/gggenes, accessed on 19 May 2025).

### 2.6. OfMADS Protein Interaction Network Analysis

The protein–protein interaction (PPI) network of OfMADS proteins was predicted utilizing the AraNet2 (https://www.inetbio.org/aranet/, accessed on 19 May 2025) database, with the analysis restricted to interactions exhibiting weight scores > 4. Functional annotation of all proteins within the PPI network was performed using the EggNOGmapper (http://eggnog-mapper.embl.de/, accessed on 19 May 2025) database [49,59]. The network visualization was conducted employing the R package ggraph (v2.2.1) (https://github.com/thomasp85/ggraph, accessed on 19 May 2025).

### 2.7. Total RNA Extraction and RNA-Seq

Roots, stems, leaves, and flowers of *O. fragrans* ‘Liuye Jingui’ were collected from the Huazhong Agricultural University campus (30°29′ N, 114°21′ W). For flowering stage-specific samples, tissues were harvested at six distinct phases: bolting stalk stage, early flowering stage, pre-flowering stage, full flowering stage, post-flowering stage, and shedding stage, with three biological replicates per stage [35]. All samples were immediately frozen in liquid nitrogen after collection and stored at −80 °C until RNA extraction. Total RNA was isolated using the RNAprep Pure Plant Kit (Tiangen, Beijing, China) following the manufacturer’s protocol. RNA quality and integrity were assessed using a NanoDrop 2000 spectrophotometer (Thermo Fisher Scientific, Waltham, MA, USA) and agarose gel electrophoresis, with only RNA samples exhibiting an RNA Integrity Number (RIN) ≥ 8.0 used for subsequent analysis. cDNA libraries were constructed using the TruSeq Stranded Total RNA Library Prep Kit (Illumina, San Diego, CA, USA) and sequenced on the Illumina NovaSeq 6000 platform (Illumina, San Diego, CA, USA). Clean reads were aligned to the *O. fragrans* reference genome using Hisat2 (v2.2.0), and transcript assembly was performed with StringTie (v2.1.5). Gene expression levels were quantified as Fragments Per Kilobase of transcript per Million mapped reads (FPKM) using FeatureCounts (v1.6.4).

### 2.8. Metabolomics and Data Analysis

Widely targeted metabolomics analysis was performed on three independent biological replicates of *O. fragrans* samples collected at six flowering stages: bolting stalk stage, early flowering stage, pre-flowering stage, full flowering stage, post-flowering stage, and shedding stage. All samples were immediately frozen in liquid nitrogen after collection, followed by freeze-drying using a vacuum freeze-dryer. The freeze-dried samples were ground into powder using a tissuelyser (64L, Jingxin, Shanghai, China) with zirconia beads at 50 Hz for 1 min. Metabolite extraction was carried out by weighing 100 mg of powder into 1.2 mL of 70% aqueous methanol. The mixture was vortexed for 30 s every 30 min, totaling 6 times, and then stored in a 4 °C refrigerator overnight. After centrifugation at 12,000 rpm for 10 min, the supernatants were filtered through a 0.22 μm pore size filter prior to UPLC-MS/MS analysis. The UPLC separation was performed on an Agilent SB-C18 column (1.8 μm, 2.1 mm × 100 mm, Santa Clara, CA, USA) with an injection volume of 2 μL, a flow rate of 0.4 mL/min, and a column temperature of 40 °C. The mobile phases consisted of water containing 0.04% acetic acid (phase A) and acetonitrile containing 0.04% acetic acid (phase B), with a gradient elution program: 5% B to 95% B over 11 min, maintaining 95% B from 11 to 12 min, decreasing to 5% B from 12 to 12.1 min, and holding 5% B from 12.1 to 14 min. Mass spectrometry detection was conducted using a system equipped with linear ion trap (LIT) and triple quadrupole (QQQ) scans. The ion spray voltages were set to 5500 V (positive ion mode) and 4500 V (negative ion mode). Other MS parameters were as follows: gas source I (GSI) = 50 psi, gas source II (GSII) = 60 psi, curtain gas (CUR) = 25 psi, and source temperature = 550 °C. Qualitative analysis of metabolites was performed by matching primary and secondary mass spectral data against the self-built MetWare (https://cloud.metware.cn/, accessed on 19 May 2025) database and public metabolite databases. Quantitative analysis was carried out using multiple reaction monitoring (MRM) mode.

### 2.9. Correlation Analysis

To quantify the relationships between *OfMADS* gene expression (transcriptome data) and metabolite accumulation (metabolome data), Pearson correlation analysis was performed using the R package ‘corrplot’ (v0.92). The correlation coefficient (r) and statistical significance (*p*-value) were calculated for each gene-metabolite pair. Pairs with |r| > 0.8 and *p* < 0.05 were defined as “strongly correlated” and retained for subsequent analysis.

## 3. Results

### 3.1. Genome-Wide Identification of the OfMADS Genes

**Figure 1:**
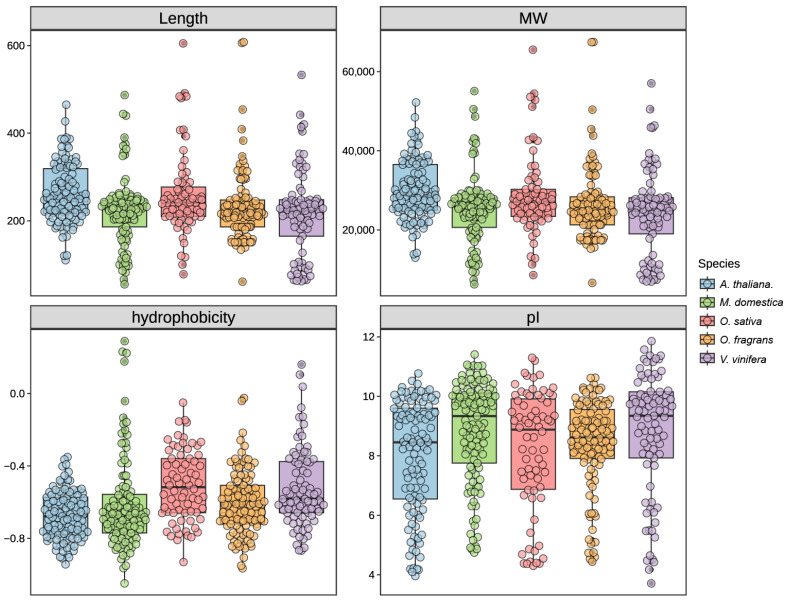
Characteristics of *MADS-box* family members in *A. thaliana*, *M. domestica*, *O. sativa*, *O. fragrans* and *V. vinifera*. The four figures represent protein length, molecular weight, hydrophilicity, and isoelectric point, respectively. Colors indicate different species: blue for *A. thaliana*, green for *M. domestica*, pink for *O. sativa*, orange for *O. fragrans*, and purple for *V. vinifera*.

In this study, a comprehensive analysis of five plant genomes revealed a total of 478 *MADS*-domain genes, with each genome containing between 70 and 113 genes. Specifically, 107 in *A. thaliana*, 113 in *M. domestica*, 70 in *O. sativa*, 107 genes were identified in *O. fragrans*, and 81 in *V. vinifera*, utilizing both HMMER (v3.2.1) and BLAST (v2.9.0+) software. Following the identification of these gene members, we conducted a detailed characterization of each candidate’s sequence attributes, including sequence length, molecular weight, theoretical isoelectric point (pI), and hydrophobicity (Figure 1, Supplementary Data S1). The predicted MADS proteins exhibited a range of 55 to 608 amino acids, molecular weights spanning 6.2 to 67.4 kDa, and theoretical pI values between 3.71 and 11.86. Additionally, the mean hydrophobicity indices of these proteins varied from −1.05 to 0.29. These results indicate that *MADS-box* genes perform various important functions in different plants.

### 3.2. Phylogenetic Analysis of the OfMADS

**Figure 2:**
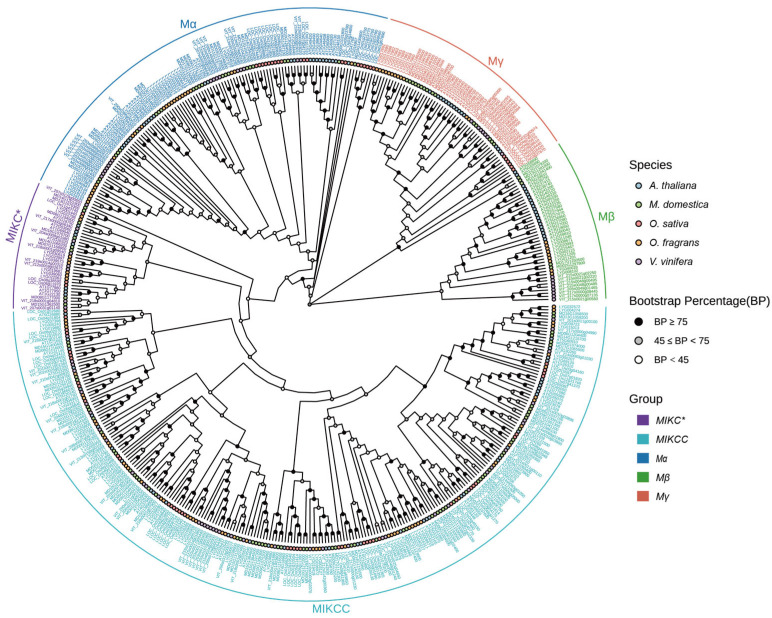
The evolutionary examination of the 478 MADS-box protein sequences identified from 5 species were conducted. These 478 MADS-box proteins were categorized into four distinct subgroups, labeled MIKC*, *MIKCC*, *Mα*, *Mβ* and *Mγ*. The evolutionary phylogeny was analyzed utilizing IQ-TREE software (2.0.3), employing the maximum likelihood (ML) method with bootstrap values derived from 1000 replicates. The phylogenetic tree was visualized through the use of the R package ggtree (3.10.0).

To elucidate the phylogenetic relationships of OfMADS proteins, a comprehensive phylogenetic tree was constructed utilizing 478 *MADS*-domain sequences. The analysis delineated five distinct subgroups: MIKC* (23 members), *MIKCC* (238 members), *Mα* (108 members), *Mβ* (43 members), and *Mγ* (55 members), with *MIKCC* emerging as the most expansive lineage (Figure 2), indicative of its evolutionary proliferation. Notably, with the exception of *O. sativa*, the member counts within the MIKC*, *Mβ*, and *Mγ* subgroups remained relatively stable across the other four species, underscoring significant divergence between dicots and monocots and suggesting that these subgroups may have evolved distinct functional roles during dicot flower development. Conversely, the *MIKCC* subgroup demonstrated only minor variations in member counts across all five species, suggesting a conserved functional role for this lineage. To substantiate the multi-species phylogeny, a phylogenetic tree was reconstructed solely utilizing OfMADS protein sequences. The topology and subgrouping observed in the Osmanthus fragrans-specific tree were entirely consistent with those obtained from the multi-species analysis, thereby corroborating the precision of our phylogenetic inference (Supplement Figure S1).

### 3.3. Conserved Motifs, Conserved Domain and Gene Structure of the OfMADS

**Figure 3:**
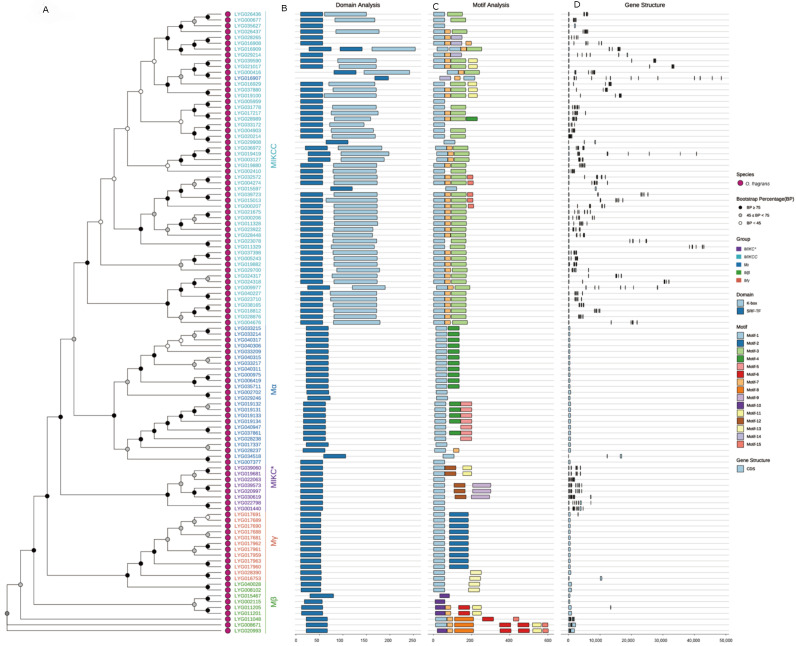
Analysis of phylogenetic relationships, conserved domains, motifs, and gene structures related to MADS-box in *O. fragrans*. (**A**) Phylogenetic tree of MADS proteins using IQ-TREE (method: maximum likelihood; bootstrap values: 1000 iterations); (**B**) Conserved domains in MADS protein sequences; (**C**) Distribution of 15 conserved motifs in MADS-box proteins; (**D**) Gene structure of *MADS-box* gene.

The diversity of protein and gene architectures offers crucial insights into the evolutionary pathways of gene families. In line with this, a systematic analysis was conducted on the conserved domains, protein motifs, and gene structures of the *OfMADS* members (Figure 3A). Domain annotation revealed that all OfMADS proteins possess at least one K-box domain, whereas the SRF-TF domain is restricted to the *MIKCC* subgroup, suggesting an expanded functional repertoire. Conversely, the *Mα*, *Mβ*, *Mγ*, and MIKC* subfamilies each contain only the K-box domain, indicating a closer evolutionary relationship among these four lineages (Figure 3B).

Conserved motifs play a crucial role in protein functionality. Through the application of the MEME suite, we identified 297 motif occurrences within 107 OfMADS proteins, which were consolidated into 15 consensus types (Motif-1–15). Notably, Motif 1 is present in nearly all OfMADS proteins, serving as a defining characteristic of the family. Additionally, certain motifs are confined to specific subgroups: Motif-3 in *MIKCC*, Motif-4 in *Mα*, Motif-5 in a subset of *Mα*, Motif-12 in MIKC*, Motif-6 in *Mβ*, and Motif-2 in *Mγ*. This specificity highlights both their high degree of conservation and their utility in delineating subgroup boundaries (Figure 3C). These subgroup-specific motifs likely contribute to the distinct biological functions of each lineage.

Gene structure analysis revealed that *OfMADS* genes contain between one and fourteen exons, with *LYG016907* has the maximum of 14 exons, while members of the Mα subfamily predominantly exhibit a single-exon architecture, whereas the *MIKCC* and MIKC* subfamilies generally display more complex exon-intron organizations. Even among genes with the same number of exons, there is considerable variation in exon lengths and arrangements. Furthermore, genes that are phylogenetically clustered tend to have more similar gene structures and overall lengths (Figure 3D).

### 3.4. Gene Location and Gene Duplication Events Analysis of OfMADS Genes

**Figure 4:**
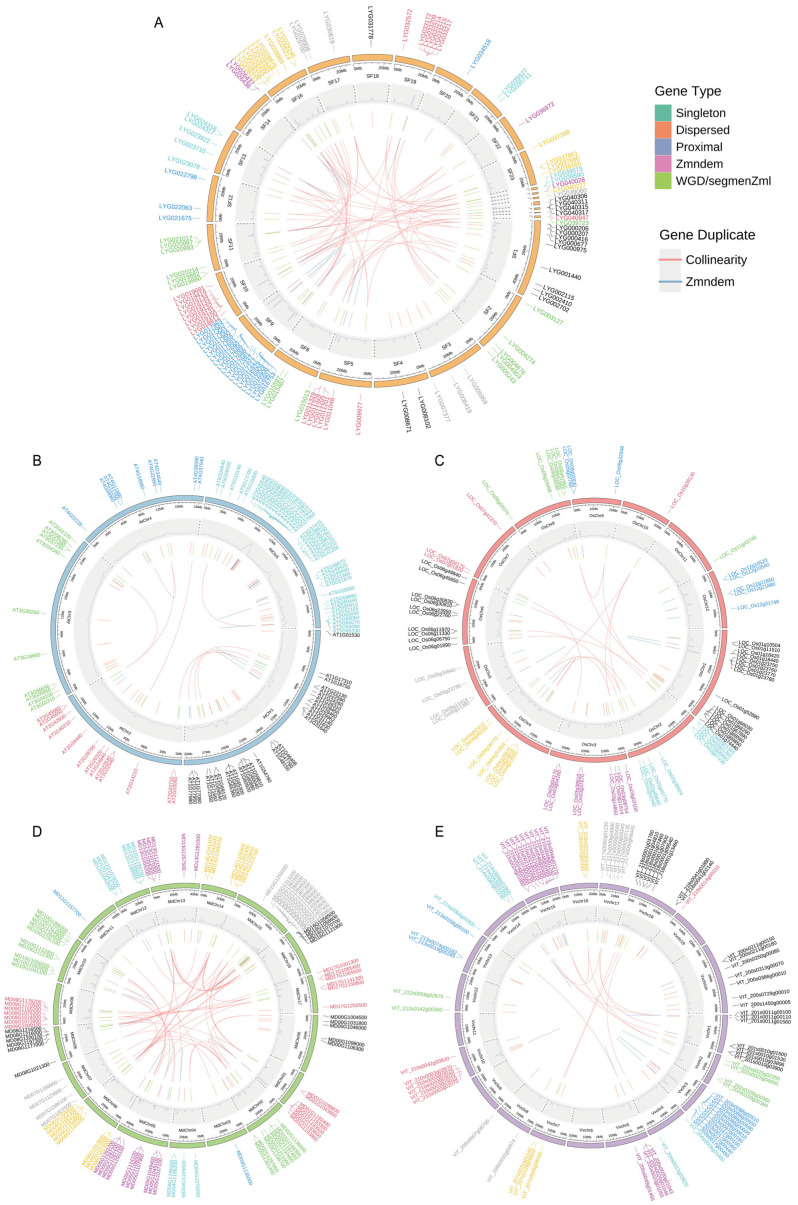
The chromosome location and duplicated gene pair of *MADS-box* genes in five species. (**A**) *O. fragrans*; (**B**) *A. thaliana*; (**C**) *O. sativa*; (**D**) *M. domestica* and (**E**) *V. vinifera*, respectively. The duplicate gene types were displayed in different colors. WGD and TD events are shown in orange and blue, respectively.

To determine the chromosomal distribution of *OfMADS* genes, we extracted their physical locations from the GFF annotation file. A total of 96 *OfMADS* genes were allocated to scaffolds 1–17, while 11 genes (*LYG039573–LYG040947*) were mapped to unanchored fragments (Figure 4). Although each primary scaffold of *O. fragrans* contains *OfMADS* loci, their distribution is notably uneven: Superscaffold 9 contains the largest number of genes (59 genes), followed by superscaffold 1 (52 genes), whereas superscaffolds 3, 20, and 21 each harbor only three genes (Figure 4). Tandem duplication (TD) and whole-genome duplication (WGD) are the primary mechanisms driving gene-family expansion. In *O. fragrans*, *A. thaliana*, *O. sativa*, *M. domestica*, and *V. vinifera*, we identified 19, 4, 5, 13, and 8 TD events, respectively, along with 60, 9, 15, 66, and 13 WGD events. These duplicated genes constitute 86.9%, 22.4%, 42.9%, 82.3%, and 40.7% of the total MADS-box repertoire in the five species (Figure 4A–E). Collectively, these findings suggest that WGD has been the predominant driver of *MADS-box* gene family expansion, with TD also playing a significant role in the evolutionary process of these species.

Additionally, the Ka, Ks, and Ka/Ks ratios for all duplicated *MADS-box* gene pairs were calculated using the KaKs_Calculator to evaluate the selective pressures experienced during gene duplication events (Supplement Figure S2). In *O. fragrans*, the gene pairs *LYG017689–LYG017688* and *LYG030632–LYG021017* exhibited Ka/Ks ratios greater than 1; similarly, in *O. sativa*, the pair *LOC_Os01g23770–LOC_Os01g23760*, and in Malus domestica, the pair *MD08G1197300–MD08G1197200* also surpassed this threshold (Ka/Ks = 1). All other duplicated gene pairs demonstrated Ka/Ks ratios less than 1, suggesting that purifying selection has been the predominant force acting on the *MADS-box* gene family. The four gene pairs with Ka/Ks ratios exceeding 1 are likely subject to positive selection, highlighting their potential importance in the evolutionary processes of these species.

### 3.5. Duplicated Gene Analysis

**Figure 5:**
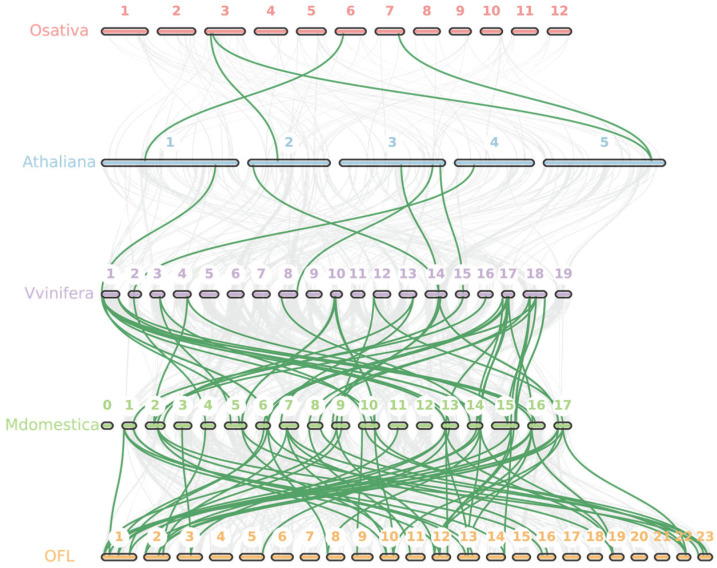
Syntenic analysis of *MADS-box* genes between *O. sativa*, *A. thaliana*, *V. vinifera*, *M. domestica*, and *O. fragrans*, respectively. The collinear blocks and *MADS-box* homologous genes pairs were shown by gray and green lines, respectively. The different numbers represent different chromosome numbers in different species.

To elucidate the evolutionary dynamics of the *MADS-box* gene family across the five species under investigation, we conducted an interspecific synteny analysis. The syntenic blocks were predominantly located on scaffolds 1, 2, 10, 12, 13, and 23 of *O. fragrans* (Figure 5). Our analysis identified six syntenic *MADS-box* gene pairs between *A. thaliana* and *V. vinifera*, 59 pairs between *M. domestica* and *O. fragrans*, 52 pairs between *V. vinifera* and *M. domestica*, and only 4 pairs between *O. sativa* and *A. thaliana* (Figure 5). These conserved loci likely originate from a common ancestor. Notably, the dicot species—*M. domestica*, *O. fragrans*, and *V. vinifera*—exhibit extensive synteny, with more than 50 gene pairs, whereas the monocot *O. sativa* and the dicot *A. thaliana* share very few syntenic pairs (four pairs). This pattern highlights a closer evolutionary relationship and conserved functional roles of *MADS-box* genes among dicots, particularly in relation to floral development and flowering processes.

### 3.6. Analysis of Promoter Elements

**Figure 6:**
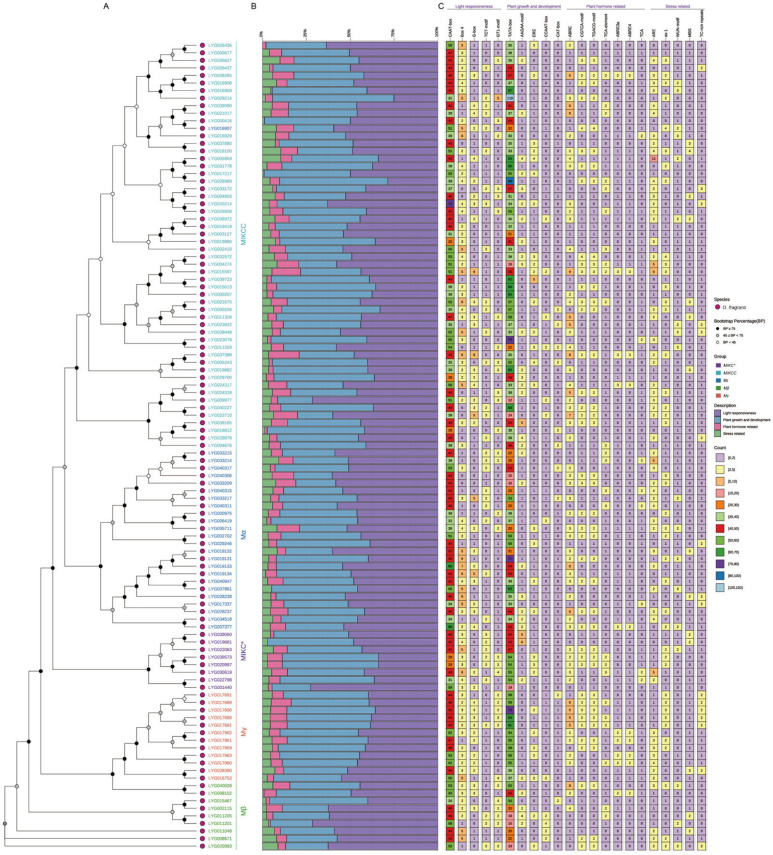
Cis-elements analysis of MADS-box genes in *O. fragrans*. (**A**) Phylogenetic tree of MADS-box proteins using IQ-TREE (method: maximum likelihood; bootstrap values: 1000 iterations); (**B**) Bar chart illustrating the summary of cis-acting elements, with colors denoting various functional categories; (**C**) Distribution of cis-acting element counts, with colors indicating different count ranges.

Gene transcription is modulated through the interaction between cis-acting elements and transcription factors. In order to investigate the potential roles of *OfMADS* family members in the growth, development, and responses of *O. fragrans* to hormonal, biotic, and abiotic stimuli, we employed the PlantCARE online tool to predict cis-acting elements within the 2.0 kb promoter region upstream of each *MADS-box* gene. Our analysis identified a total of 22 distinct types of elements (Figure 6A), categorized as follows: five light-responsive elements, including the *CAAT-box*, *Box-4*, *G-box*, *TCT-motif*, and *GT1-motif*; five growth- and development-related elements, namely the *TATA-box*, *AAGAA-motif*, *ERE*, *CCAAT-box*, and *CAT-box*; seven hormone-responsive elements, which are *ABRE*, *CGTCA-motif*, *TGACG-motif*, *TCA-element*, *ABRE3a*, *ABRE4*, and *TCA*; and five stress-responsive elements, including *ARE*, *as-1*, *WUN-motif*, *MBS*, and *TC-rich* repeats.

Quantitative analysis revealed that light- and development-related elements are the most prevalent in *OfMADS* promoters, followed by hormone-responsive elements, with stress-responsive elements being the least common (Figure 6B). Within the light-responsive elements, the *CAAT-box* was the most frequently occurring, followed by *Box-4*. In terms of development-related elements, the *TATA-box* was the most prevalent, followed by the *AAGAA-motif*. This distribution pattern was consistently observed across all members of the *MADS-box* family (Figure 6C). These findings suggest that *OfMADS* promoters are evolutionarily enriched with elements that regulate light and growth/development, indicating a significant role for MADS proteins in facilitating light-dependent regulation during the growth and development of *O. fragrans*.

### 3.7. Interaction Network of MADS-Box Proteins

**Figure 7:**
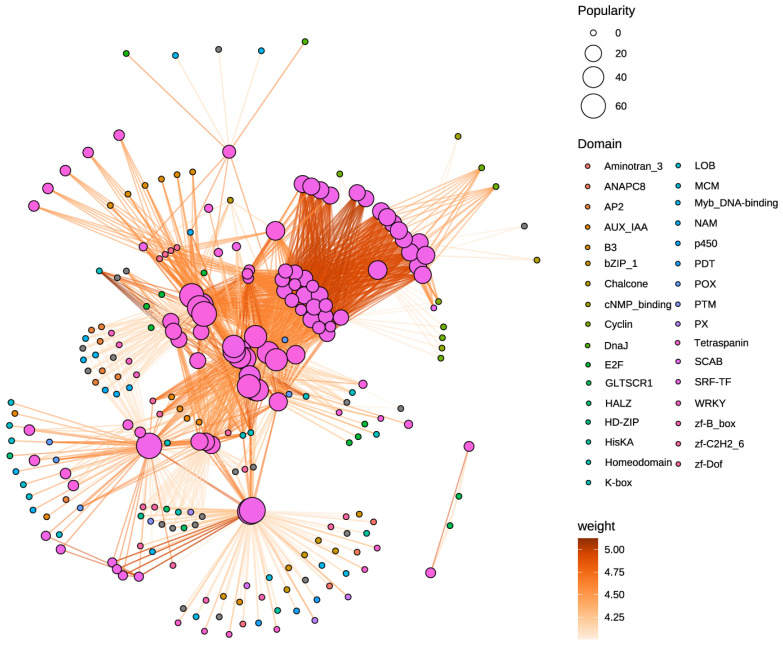
Protein–protein interaction (PPI) network of MADS-box proteins in *O. fragrans*. The colors of the node indicate different domain, while the colors of edges correspond to the interaction weights.

To further elucidate the functions and regulatory network of the *MADS-box* gene family in *O. fragrans*, we conducted a prediction and construction of its protein–protein interaction (PPI) network (Figure 7). This PPI network included 245 proteins. Network analysis revealed extensive intrafamily interactions among MADS proteins, indicating functional synergy and providing new insights into their specificity and redundancy within particular biological pathways or cellular processes.

Furthermore, MADS-box proteins were found to interact with several transcription factors, including *B3*, *SCAB*, *WRKY*, and *MYB*. Previous studies have demonstrated that the WRKY and MYB families play pivotal roles in plant growth, development, and floral organogenesis, suggesting that *MADS-box*, *WRKY*, and *MYB* may cooperatively regulate flower development in *O. fragrans.* These findings lay a robust foundation for subsequent functional validation and mechanistic investigation, highlighting their significant research value.

### 3.8. GO and KEGG Enrichment Analysis of the OfMADS Genes

**Figure 8:**
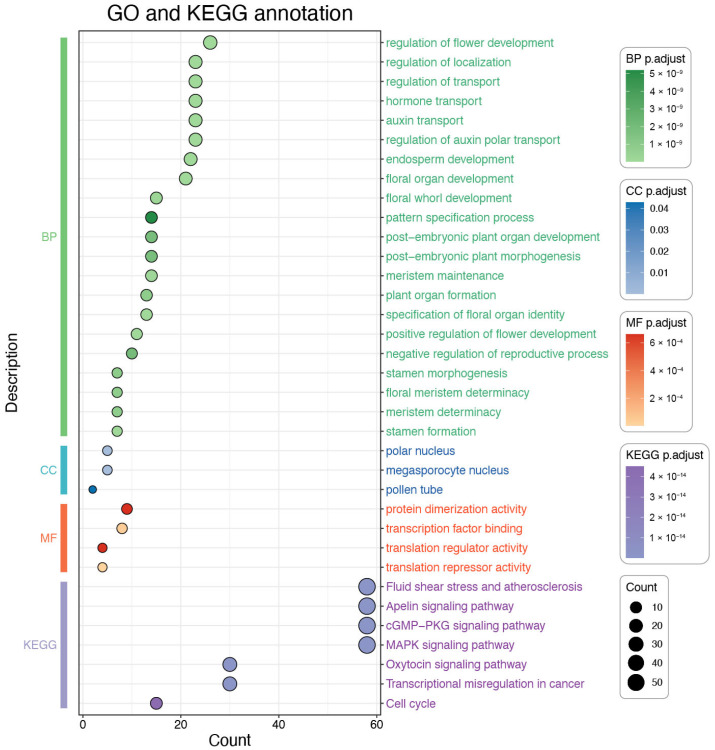
The enrichment analysis of Gene Ontology (GO) and Kyoto Encyclopedia of Genes and Genomes (KEGG) pathways for MADS-box genes in *O. fragrans*. The GO terms are systematically classified into three categories: Biological Process (BP), Cellular Component (CC), and Molecular Function (MF). The vertical axis illustrates the annotated GO terms, whereas the horizontal axis indicates the number of genes associated with each GO term. The categories BP, CC, and MF are depicted in green, blue, and red, respectively, while KEGG pathways are represented in purple.

To further investigate the biological functions associated with *OfMADS* genes, we conducted Gene Ontology (GO) and KEGG pathway enrichment analyses on a set of 107 *OfMADS* genes (Figure 8). The GO terms were categorized into three distinct groups: biological processes (BP), cellular components (CC), and molecular functions (MF). Within the BP category, *OfMADS* genes exhibited significant enrichment in processes such as the regulation of flower development, regulation of localization, hormone transport, auxin transport, and floral organ development. In the CC category, notable enrichment was observed in the polar nucleus, megasporocyte nucleus, and pollen tube. For the MF category, the enriched functions included protein dimerization activity, transcription factor binding, translation regulator activity, and translation repressor activity. The KEGG pathway analysis identified significant enrichment in pathways related to fluid shear stress and atherosclerosis, the MAPK signaling pathway, the Apelin signaling pathway, and the cell cycle. Collectively, these findings suggest that *OfMADS* genes are predominantly involved in flower development, hormone transport, and signal transduction, indicating their potential critical roles in the developmental processes of *O. fragrans*.

### 3.9. Transcriptome Data Analysis of Different Tissues

**Figure 9:**
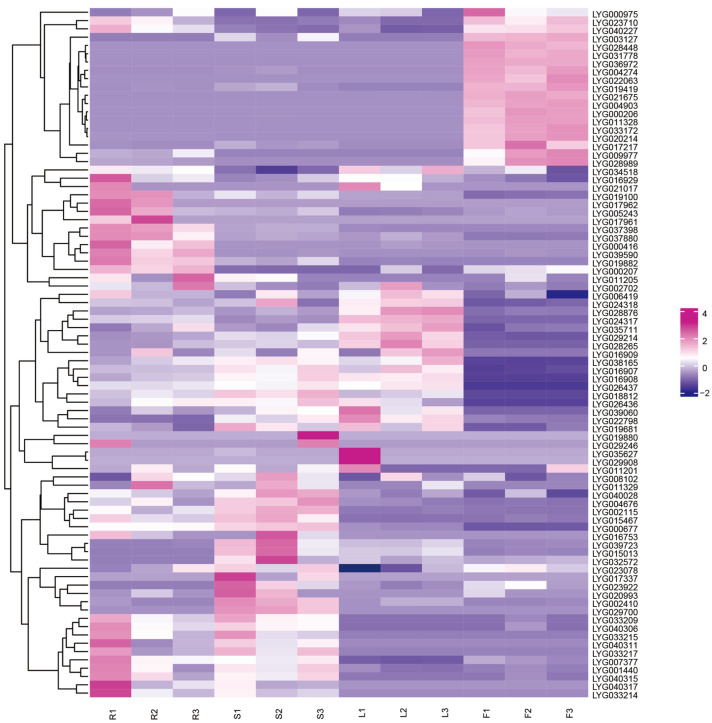
Transcriptome data analysis of different tissues in *O. fragrans*. Purple indicates high expression levels, and blue indicates low expression levels. R represents root, S represents stem, L represents leaf, and F represents flower. Each tissue has three biological replicates.

To investigate the roles of *MADS-box* genes in different tissues, the transcriptional levels of 83 *MADS-box* genes in roots, stems, leaves, and flowers were visualized via a heatmap. The results revealed that 19 genes were highly expressed in flowers, among which 16 genes (i.e., *LYG000206*, *LYG003127*, *LYG004274*, *LYG004903*, *LYG009977*, *LYG011328*, *LYG017217*, *LYG019419*, *LYG020214*, *LYG021675*, *LYG022063*, *LYG028448*, *LYG028989*, *LYG031778*, *LYG033172*, and *LYG036972*) exhibited specific high expression exclusively in flowers (Figure 9). Additionally, all these genes are enriched with *CAAT-box* and *TATA-box*, suggesting that they may be involved in the flowering process or reproductive processes of flowers (Figure 6).

### 3.10. Transcriptome Analysis of Different Flowering Stages

**Figure 10:**
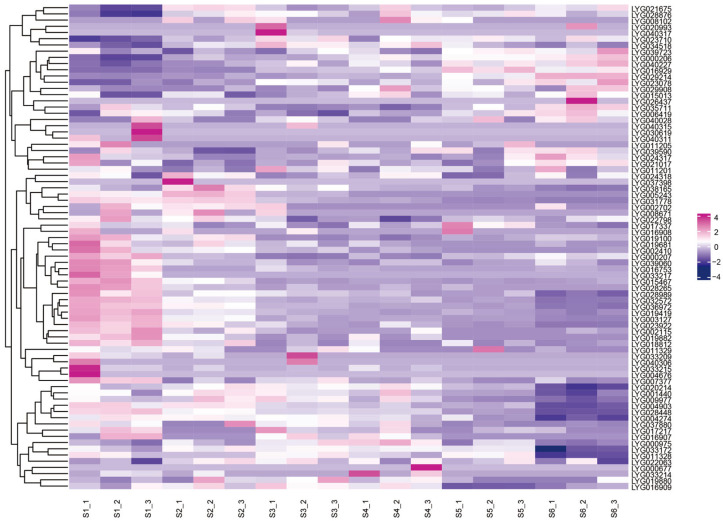
Transcriptome data analysis of different flowering stages in *O. fragrans*. The heatmap illustrates the gene expression patterns across six flowering stages of Osmanthus fragrans, with three biological replicates for each stage. S1, S2, S3, S4, S5, and S6 correspond to the bolting stalk stage, early flowering stage, pre-flowering stage, full flowering stage, post-flowering stage, and shedding stage, respectively. The color gradient, ranging from blue (low expression) to purple (moderate expression) and pink (high expression), reflects the differences in gene expression levels.

To further identify genes associated with the flowering process, we analyzed the transcriptome profiles of *O. fragrans* across six flowering stages, namely the bolting stalk stage, early flowering stage, pre-flowering stage, full flowering stage, post-flowering stage, and shedding stage (Figure 10). A total of 78 genes showed differential expression across different flowering stages (Figure 10).

These 78 genes could be roughly classified into four groups: the first group exhibited a continuously upregulated trend throughout the entire flowering process; the second group showed a trend of first decreasing and then increasing during flowering; the third group displayed a continuously downregulated trend over the whole flowering period; and the fourth group presented a trend of first increasing and then decreasing during flowering (Figure S3; Table S2). Among them, 16 flower-specific highly expressed genes showed differential changes across the six flowering stages: *LYG000206* presented a continuous upward trend; *LYG003127*, *LYG004903*, *LYG019419*, *LYG028448*, *LYG028989* and *LYG004274* showed a continuous downward trend; *LYG022063*, *LYG020214*, *LYG009977*, and *LYG004903* exhibited a trend of first increasing and then decreasing; *LYG017217* and *LYG011328* displayed a trend of first decreasing, then increasing, and then decreasing again (Figure 10).

This indicates that different *MADS-box* genes play distinct roles in the flowering process, and, in particular, the genes that first increase and then decrease during flowering may be closely related to floral scent, flower color, and other traits.

### 3.11. Integrated Transcriptome and Metabolome Analysis of Different Flowering Stages

To further investigate the physiological mechanisms underlying different flowering stages of *O. fragrans*, we also performed metabolite detection on samples across six flowering stages, with a total of 531 metabolites detected (Figure S3). Subsequently, we calculated the correlations between *MADS-box* genes and 531 metabolites, screening for metabolites with a correlation coefficient greater than 0.8, which ultimately yielded 1749 strongly correlated metabolite-gene pairs (Table S3). Among these, 49 MADX-BOX showed strong correlations with metabolites and 262 metabolites showed strong correlations with *MADX-BOX* genes, *LYG003127* with 106 metabolites, *LYG028448* with 102 metabolites, *LYG019419* with 99 metabolites, *LYG004903* with 53 metabolites, *LYG009977* with 8 metabolites and *LYG020214* with 22 metabolites.

Notably, these genes contain multiple *CAAT-box* and *TATA-box* binding domains, are specifically highly expressed in flowers, and are correlated with multiple metabolites such as diosmetin and jasmonic acid which generate volatile aldehydes/ketones and regulates terpene biosynthesis in *O. fragrans.*

## 4. Discussion

*MADS-box* transcription factors are significant regulatory elements that are ubiquitously present and highly conserved across eukaryotic organisms, and they have been demonstrated to play a pivotal role in plant growth and development [60,61,62,63,64]. In recent years, the biological functions of *MADS-box* genes in floral development have garnered increasing attention from plant biologists [65,66,67,68]. As a pivotal class of transcription factors, *MADS-box* genes are integral to the regulation of floral organ differentiation, flowering time, and reproductive development [69,70,71]. Prior research has demonstrated considerable variation in the number of *MADS* genes among plant species, likely attributable to factors such as genome size, polyploidy events, and species-specific developmental requirements [5,17,34,60]. Notably, 107 *MADS-box* genes have been identified in *O. fragrans* with an uneven chromosomal distribution, with some gene clusters localized to specific chromosomal regions, suggesting that gene duplication events may have contributed to their expansion. In comparison to model organisms such as *A. thaliana* and *O. sativa*, the *MADS-box* gene families are typically more abundant in dicotyledonous species than in monocotyledonous species. For example, both *A. thaliana* and *M. domestica* possess a greater number of *MADS-box* genes than *O. sativa*, suggesting that this gene family has experienced more extensive expansion in dicots [33,72]. This expansion may be attributed to the more intricate floral structures and varied reproductive strategies observed in dicots [73].

This study identified multiple *OfMADS* genes closely associated with floral development, and their functions exhibit both conservation and divergence compared to previously characterized floral development-related *MADS-box* genes in *O. fragrans* and other species. On the one hand, several *OfMADS* genes share conserved roles with known floral regulators: for example, the flower-specific highly expressed genes *LYG003127*, *LYG028448*, and *LYG019419*—enriched with *CAAT-box* and *TATA-box* elements—show expression patterns and cis-regulatory features consistent with the B-class gene *OfGLO1* identified by Zeng et al. (2021) [39]. Both *OfGLO1* and these newly identified *OfMADS* genes are highly expressed in floral organs and implicated in floral organ identity, reflecting conservation of B-class gene functions in specifying stamen and petal development. Similarly, *LYG004903* and *LYG022063*, which exhibit a “first increasing then decreasing” expression trend across flowering stages, share functional similarity with *OfAP1-a*, a gene that modulates floral transition and petal number in *O. fragrans* ‘Sijigui’—as both are involved in coordinating floral maturation processes [40].

The pronounced disparities in *MADS-box* gene copy numbers between monocots and dicots imply divergent evolutionary pathways and functional adaptations of this gene family within these two groups. Moreover, a comparative analysis of the ancient tetraploid Vitis vinifera and other dicotyledonous species, such as *O. fragrans*, *A. thaliana*, and *M. domestica*, demonstrated that these latter species possess a greater number of *MADS-box* genes than grapevine. This observation suggests that the *MADS-box* gene family has undergone further expansion throughout the evolution of dicots [74,75,76]. Collectively, these findings not only emphasize the pivotal role of *MADS-box* genes in influencing plant morphology but also highlight their functional adaptability in enabling plants to thrive in diverse ecological environments and reproductive contexts.

The cis-acting element analysis of the promoter regions of Osmanthus fragrans *MADS-box* genes demonstrated a significant enrichment of *CAAT-box* elements, which are associated with light responsiveness, and *TATA-box* elements, which are involved in fundamental transcriptional activity and plant growth and development. These results imply that the expression of *O. fragrans MADS-box* genes may be modulated by light signals and could play a role in the precise regulation of developmental processes at the transcriptional level. Further predictions regarding protein–protein interactions indicated that Osmanthus fragrans MADS-box proteins have the potential to interact with various key regulatory proteins, particularly transcription factors such as *WRKY* and *MYB*-known regulators of floral organogenesis and secondary metabolism [60,77,78], implying complex regulatory modules that coordinate flower development with scent biosynthesis, a key horticultural trait of *O. fragrans*.

Gene duplication events have been pivotal in driving the functional diversification of the *MADS-box* gene family in plants [74,75,76]. For example, the duplicated gene pair *LYG011328* and *LYG011329* demonstrated distinct tissue-specific expression patterns, with one gene being upregulated in flowers and the other in leaves. This observation underscores functional divergence following gene duplication. Consequently, such duplication events have not only expanded the number of *MADS-box* family members but have also facilitated their functional specialization and diversification [73]. These observations align with findings in other species, including *A. thaliana* and *M. domestica*, where *MADS-box* genes demonstrate significant evolutionary conservation and are integral to growth and development [79]. Moreover, gene duplication events are widely acknowledged as crucial mechanisms for expanding and refining the functional repertoire of the MADS family across species [55]. Collectively, this study establishes a theoretical framework for comprehending the structural characteristics, expression patterns, and evolutionary dynamics of the *MADS-box* gene family in *O. fragrans*, while also providing valuable insights into the functional evolution of *MADS-box* genes throughout the plant kingdom.

Transcriptome profiling across six flowering stages (from bolting to shedding) revealed 78 differentially expressed *OfMADS* genes, with distinct expression patterns. Notably, genes showing a “first increasing then decreasing” trend (e.g., *LYG022063*, *LYG004903*) may regulate peak floral traits, such as scent emission or pigment accumulation, which are most prominent during the full flowering stage. This aligns with GO enrichment results linking *OfMADS* genes to “floral organ development”, suggesting that these *OfMADS* genes may bridge transcriptional regulation of flowering with secondary metabolism-critical for pollinator attraction and ornamental value.

The findings of this study provide practical strategies for regulating *O. fragrans* flowering time in horticultural production, enabling both flowering time advancement andflowering duration extension. For advancing flowering, we can target *OfMADS* genes with repressive roles in floral transition, drawing on the conserved function of *Dormancy associated MADS-box* (*DAM*) genes (flowering repressors) in *Malus pumila* Mill. (apple) [80]. For instance, *LYG003127*—highly expressed in early flowering stages and negatively correlated with floral maturation—exhibits functional similarity to peach *DAM* genes (whose deletion reduces chilling requirements [81]). Using CRISPR-Cas9 to knock out *LYG003127* could reduce its repressive effect on downstream floral promoters, potentially advancing blooming. Alternatively, for non-transgenic approaches, since *CAAT-box* elements in *OfMADS* promoters mediate light responsiveness, extending short-day treatments during the bolting stage can activate *LYG000206* expression, accelerating floral transition.

To extend flowering duration, we can leverage *OfMADS* genes involved in floral senescence regulation. For example, *LYG022063*—whose expression peaks at full bloom and declines during petal shedding. Modulating *LYG022063* expression via promoter editing might could delay its downregulation: low auxin concentrations has been shown to inhibit floral senescence in *O. fragrans* [82], and introducing auxin responsiveness to *LYG022063* might could prolong its role in maintaining floral organ integrity. For further enhancing the efficacy of these strategies, combining *OfMADS* modulation with WRKY transcription factor co-expression can strengthen the regulatory network coordinating flowering and senescence, as *WRKY-MADS* interactions have been shown to enhance secondary metabolism and stress tolerance [60], which indirectly supports longer floral longevity.

These strategies integrate the conserved *MADS-box* regulatory mechanisms derived from model plant species with the species-specific genetic characteristics of *O. fragrans*, thereby enhancing the efficiency of practical breeding programs while safeguarding the key ornamental traits of this species [82,83]. By prioritizing *OfMADS* genes with well-characterized functions in floral transition and senescence, horticultural researchers and practitioners can precisely modulate the flowering phenology of *O. fragrans* to align with market demands—for instance, advancing the blooming period to coincide with early spring festivals or prolonging the flowering duration to augment its landscape application value.

## 5. Conclusions

In summary, our study identified 107 *OfMADS* genes in *O. fragrans*, with protein lengths ranging from 61 to 608 amino acids. These genes were phylogeneticlly classified into five subgroups: MIKC*, *MIKCC*, *Mα*, *Mβ*, and *Mγ*, within conserved motifs within each subgroup but notable differences among subfamilies. Tandem and whole-genome duplications likely contributed to the expansion of *MADS-box* gene family in *O. fragrans*. Cis-element analysis suggested the involvement of these genes in hormone signaling pathways and plant growth and development. The presence of light- and growth-related promoter elements, along with their interactions with *WRKY/MYB* factors, highlighted their integration into developmental and metabolic regulatory networks. Transcriptome data revealed 16 flower-specific *OfMADS* genes, with 78 showing stage-specific expression across six flowering phases, some of which exhibited a “first increasing then decreasing” patterns associated with peak floral traits. Integrated metabolomics further demonstrated strong associations between *OfMADS* genes and metabolites, hinting at their potential role in scent-related terpene biosynthesis. These findings significantly enhance our understanding of the *MADS-box* gene family in *O. fragrans*, providing a valuable foundation for further in-depth functional characterization of these genes. In the future, this knowledge can be applied to genetically manipulate the flowering time and floral traits of *O. fragrans*, potentially improving its ornamental value in horticultural and landscape settings.

## Data Availability

The original contributions presented in this study are included in the article. Further inquiries can be directed to the corresponding author.

## Supplementary Materials

The following supporting information can be downloaded at: https://www.mdpi.com/article/10.3390/cimb47100819/s1.

## References

[1] Passmore S., Maine G.T., Elble R., Christ C., Tye B.-K. (1988). Saccharomyces cerevisiae protein involved in plasmid maintenance is necessary for mating of MATα cells. J. Mol. Biol..

[2] Sommer H., Beltran J.-P., Huijser P., Pape H., Lönnig W.-E., Saedler H., Schwarz-Sommer Z. (1990). Deficiens, a homeotic gene involved in the control of flower morphogenesis in Antirrhinum majus: The protein shows homology to transcription factors. EMBO J..

[3] Yanofsky M.F., Ma H., Bowman J.L., Drews G.N., Feldmann K.A., Meyerowitz E.M. (1990). The protein encoded by the Arabidopsis homeotic gene agamous resembles transcription factors. Nature.

[4] Honma T., Goto K. (2001). Complexes of MADS-box proteins are sufficient to convert leaves into floral organs. Nature.

[5] Tahmasebi S., Jonoubi P., Majdi M., Majd A., Heidari P. (2025). Genome-wide characterization and expression profiling of MADS-box family genes during organ development and drought stress in Camelina sativa L. Sci. Rep..

[6] Alvarez-Buylla E.R., Pelaz S., Liljegren S.J., Gold S.E., Burgeff C., Ditta G.S., de Pouplana L.R., Martínez-Castilla L., Yanofsky M.F. (2000). An ancestral MADS-box gene duplication occurred before the divergence of plants and animals. Proc. Natl. Acad. Sci. USA.

[7] Allam G., Sakariyahu S.K., Shan B., Aung B., McDowell T., Papadopoulos Y., Bernards M.A., Hannoufa A. (2025). Aluminum Stress Response Is Regulated Through a miR156/SPL13 Module in Medicago sativa. Genes.

[8] Basak M., Chakraborty S., Kundu S., Dey S., Das M. (2025). Identification, expression analyses of APETALA1 gene homologs in Bambusa tulda and heterologous validation of BtMADS14 in Arabidopsis thaliana. Physiol. Mol. Biol. Plants.

[9] Cai Y., Yang W., Yue J., Chen J., Xing J., Yang X., Ye D., Tang C., Liu H. (2025). Isolation and Functional Characterization of the MADS-Box Gene AGAMOUS-LIKE 24 in Rubber Dandelion Taraxacum kok-saghyz Rodin. Int. J. Mol. Sci..

[10] Cheng Y., Cheng L., Hu G., Guo X., Liu Z., Lan Y. (2025). The MADS-box gene CmAP1 promotes flowering and petal development in Chinese chestnut Castanea mollissima. BMC Plant Biol..

[11] Fang J., Chun Y., Zhang F., Guo T., Ren M., Zhao J., Yuan S., Wang W., Li Y., Li X. (2025). A Novel OsMPK6-OsMADS47-PPKL1/3 Module Controls Grain Shape and Yield in Rice. Adv. Sci..

[12] Gao X., Li Y., Dai Y., Li X., Huang C., Zhang S., Li F., Zhang H., Li G., Sun R. (2025). Identification of the MADS-Box Gene Family and the Key Role of BrAGL27 in the Regulation of Flowering in Chinese Cabbage *Brassica rapa* L. ssp. pekinensis. Int. J. Mol. Sci..

[13] De Bodt S., Raes J., Van de Peer Y., Theißen G. (2003). And then there were many: MADS goes genomic. Trends Plant Sci..

[14] Kaufmann K., Melzer R., Theißen G. (2005). MIKC-type MADS-domain proteins: Structural modularity, protein interactions and network evolution in land plants. Gene.

[15] Theißen G., Melzer R., Rümpler F. (2016). MADS-domain transcription factors and the floral quartet model of flower development: Linking plant development and evolution. Development.

[16] Saedler H., Becker A., Winter K.-U., Kirchner C., Theißen G. (2001). MADS-box genes are involved in floral development and evolution. Acta Biochim. Pol..

[17] Riechmann J.L., Krizek B.A., Meyerowitz E.M. (1996). Dimerization specificity of Arabidopsis MADS domain homeotic proteins APETALA1, APETALA3, PISTILLATA, and AGAMOUS. Proc. Natl. Acad. Sci. USA.

[18] Han J.P., Wan J.N., Guan Z.L., Xu H., Wang Q.F., Wan T. (2025). The origin, evolution and diversification of MADS-box transcription factors in green plants. Plant Commun..

[19] Hu X., Zhang Y., Wang J., Du M., Yang Y., Cai J.J., Yang E. (2025). Functional diversification of the MADS-box gene family in fine-tuning the dimorphic transition of Talaromyces marneffei. mSystems.

[20] Ren L., Sun H., Dai S., Feng S., Qiao K., Wang J., Gong S., Zhou A. (2021). Identification and Characterization of MIKCc-Type MADS-Box Genes in the Flower Organs of Adonis amurensis. Int. J. Mol. Sci..

[21] Lyu K.L., Zeng S.M., Huang X.Z., Jiang C.C. (2025). Integrated Multi-Omics Reveals DAM-Mediated Phytohormone Regulatory Networks Driving Bud Dormancy in ‘Mixue’ Pears. Plants.

[22] Nobles A., Wendel J.F., Yoo M.J. (2025). Comparative Analysis of Floral Transcriptomes in Gossypium hirsutum Malvaceae. Plants.

[23] Parenicova L., De Folter S., Kieffer M., Horner D.S., Favalli C., Busscher J., Cook H.E., Ingram R.M., Kater M.M., Davies B. (2003). Molecular and phylogenetic analyses of the complete MADS-box transcription factor family in Arabidopsis: New openings to the MADS world. Plant Cell.

[24] Theissen G., Melzer R. (2007). Molecular mechanisms underlying origin and diversification of the angiosperm flower. Ann. Bot..

[25] Su Q., Tian M., Wang H., Huang Z., Awais M., Xu X., Wang L., Lai Z., Bu Z. (2025). Transcriptome analysis reveals that regulation network of the genes related to unique double flowers in tropical viviparous water lily. Sci. Rep..

[26] Sun J., Zhou Z., Meng F., Wen M., Liu A., Yu A. (2025). Characterization analyses of MADS-box genes highlighting their functions with seed development in Ricinus communis. Front. Plant Sci..

[27] Tan B., Xie Y., Peng H., Wang M., Zhu W., Xu L., Cheng Y., Wang Y., Zeng J., Fan X. (2025). Transcriptome Profiling of Spike Development Reveals Key Genes and Pathways Associated with Early Heading in Wheat-Psathyrstachys huashanica 7Ns Chromosome Addition Line. Plants.

[28] Wang J., Wang L., Usman M., Zhu J., Jiu S., Liu R., Zhang C. (2025). ABA-Insensitive 5 ABI5 Is Involved in ABA-Induced Dormancy via Activating PavCIG1/2 Expression in Sweet Cherries. Genes.

[29] Coen E.S., Meyerowitz E.M. (1991). The war of the whorls: Genetic interactions controlling flower development. Nature.

[30] Zahn L.M., Feng B., Ma H. (2006). Beyond the ABC-model: Regulation of floral homeotic genes. Adv. Bot. Res..

[31] Liu E., Zhu S., Du M., Lyu H., Zeng S., Liu Q., Wu G., Jiang J., Dang X., Dong Z. (2023). LAX1, functioning with MADS-box genes, determines normal palea development in rice. Gene.

[32] Mandel M.A., Gustafson-Brown C., Savidge B., Yanofsky M.F. (1992). Molecular characterization of the Arabidopsis floral homeotic gene APETALA1. Nature.

[33] Jack T., Brockman L.L., Meyerowitz E.M. (1992). The homeotic gene APETALA3 of Arabidopsis thaliana encodes a MADS box and is expressed in petals and stamens. Cell.

[34] Alvarez-Buylla E.R., García-Ponce B., Sánchez M.P., Espinosa-Soto C., García-Gómez M.L., Piñeyro-Nelson A., Garay-Arroyo A. (2019). MADS-box genes underground becoming mainstream: Plant root developmental mechanisms. New Phytol..

[35] Chen H., Zeng X., Yang J., Cai X., Shi Y., Zheng R., Wang Z., Liu J., Yi X., Xiao S. (2021). Whole-genome resequencing of Osmanthus fragrans provides insights into flower color evolution. Hortic. Res..

[36] Li Y., Xia H., Cushman S.A., Zhao H., Guo P., Liu Y.P., Lin N., Shang F.D. (2023). A new mechanism of flowering regulation by the competition of isoforms in Osmanthus fragrans. Ann. Bot..

[37] Wang Q., Gao G., Chen X., Liu X., Dong B., Wang Y., Zhong S., Deng J., Fang Q., Zhao H. (2022). Genetic studies on continuous flowering in woody plant Osmanthus fragrans. Front. Plant Sci..

[38] Wang Q., Chen X., Liu X., Gao G., Dong B., Wang Y., Zhong S., Deng J., Fang Q., Zhao H. (2024). OfBFT genes play an essential role in the proliferate flower formation of Osmanthus fragrans. Plant Physiol. Biochem..

[39] Zeng Z., Chen S., Xu M., Wang M., Chen Z., Wang L., Pang J. (2021). Cloning, Expression, and Tobacco Overexpression Analyses of a PISTILLATA/GLOBOSA-like (OfGLO1) Gene from Osmanthus fragrans. Genes.

[40] Liu X., Wang Q., Jiang G., Wan Q., Dong B., Lu M., Deng J., Zhong S., Wang Y., Khan I.A. (2023). Temperature-responsive module of OfAP1 and OfLFY regulates floral transition and floral organ identity in Osmanthus fragrans. Plant Physiol. Biochem..

[41] Hugouvieux V., Silva C.S., Jourdain A., Stigliani A., Charras Q., Conn V., Conn S.J., Carles C.C., Parcy F., Zubieta C. (2018). Tetramerization of MADS family transcription factors SEPALLATA3 and AGAMOUS is required for floral meristem determinacy in Arabidopsis. Nucleic Acids Res..

[42] Pinyopich A., Ditta G.S., Savidge B., Liljegren S.J., Baumann E., Wisman E., Yanofsky M.F. (2003). Assessing the redundancy of MADS-box genes during carpel and ovule development. Nature.

[43] Ditta G., Pinyopich A., Robles P., Pelaz S., Yanofsky M.F. (2004). The SEP4 gene of Arabidopsis thaliana functions in floral organ and meristem identity. Curr. Biol..

[44] Yan T., Yoo D., Berardini T.Z., Mueller L.A., Weems D.C., Weng S., Cherry J.M., Rhee S.Y. (2005). PatMatch: A program for finding patterns in peptide and nucleotide sequences. Nucleic Acids Res..

[45] Ouyang S., Zhu W., Hamilton J., Lin H., Campbell M., Childs K., Thibaud-Nissen F., Malek R.L., Lee Y., Zheng L. (2007). The TIGR Rice Genome Annotation Resource: Improvements and new features. Nucleic Acids Res..

[46] Goodstein D.M., Shu S., Howson R., Neupane R., Hayes R.D., Fazo J., Mitros T., Dirks W., Hellsten U., Putnam N. (2012). Phytozome: A comparative platform for green plant genomics. Nucleic Acids Res..

[47] Paysan-Lafosse T., Blum M., Chuguransky S., Grego T., Pinto B.L., Salazar G.A., Bileschi M.L., Bork P., Bridge A., Colwell L. (2023). InterPro in 2022. Nucleic Acids Res..

[48] Mistry J., Finn R.D., Eddy S.R., Bateman A., Punta M. (2013). Challenges in homology search: HMMER3 and convergent evolution of coiled-coil regions. Nucleic Acids Res..

[49] Camacho C., Coulouris G., Avagyan V., Ma N., Papadopoulos J., Bealer K., Madden T.L. (2009). BLAST+: Architecture and applications. BMC Bioinform..

[50] Edgar R.C. (2004). MUSCLE: Multiple sequence alignment with high accuracy and high throughput. Nucleic Acids Res..

[51] Nguyen L.-T., Schmidt H.A., Von Haeseler A., Minh B.Q. (2015). IQ-TREE: A fast and effective stochastic algorithm for estimating maximum-likelihood phylogenies. Mol. Biol. Evol..

[52] Yu G., Smith D.K., Zhu H., Guan Y., Lam T.T.-Y. (2017). ggtree: An R package for visualization and annotation of phylogenetic trees with their covariates and other associated data. Methods Ecol. Evol..

[53] Bateman A., Coin L., Durbin R., Finn R.D., Hollich V., Griffiths-Jones S., Khanna A., Marshall M., Moxon S., Sonnhammer E.L.L. (2004). The Pfam protein families database. Nucleic Acids Res..

[54] Bailey T.L., Johnson J., Grant C.E., Noble W.S. (2015). The MEME Suite. Nucleic Acids Res..

[55] Wang Y., Tang H., DeBarry J.D., Tan X., Li J., Wang X., Lee T.-h., Jin H., Marler B., Guo H. (2012). MCScanX: A toolkit for detection and evolutionary analysis of gene synteny and collinearity. Nucleic Acids Res..

[56] Thompson J.D., Gibson T.J., Higgins D.G. (2003). Multiple sequence alignment using ClustalW and ClustalX. Curr. Protoc. Bioinform..

[57] Zhang Z. (2022). KaKs_Calculator 3.0: Calculating selective pressure on coding and non-coding sequences. Genom. Proteom. Bioinform..

[58] Lescot M., Déhais P., Thijs G., Marchal K., Moreau Y., Van de Peer Y., Rouzé P., Rombauts S. (2002). PlantCARE, a database of plant cis-acting regulatory elements and a portal to tools for in silico analysis of promoter sequences. Nucleic Acids Res..

[59] Cantalapiedra C.P., Hernández-Plaza A., Letunic I., Bork P., Huerta-Cepas J. (2021). eggNOG-mapper v2: Functional annotation, orthology assignments, and domain prediction at the metagenomic scale. Mol. Biol. Evol..

[60] Abdullah-Zawawi M.R., Ahmad-Nizammuddin N.F., Govender N., Harun S., Mohd-Assaad N., Mohamed-Hussein Z.A. (2021). Comparative genome-wide analysis of WRKY, MADS-box and MYB transcription factor families in Arabidopsis and rice. Sci. Rep..

[61] Adhikari P.B., Kasahara R.D. (2024). An Overview on MADS Box Members in Plants: A Meta-Review. Int. J. Mol. Sci..

[62] Zhang Z., Zou W., Lin P., Wang Z., Chen Y., Yang X., Zhao W., Zhang Y., Wang D., Que Y. (2024). Evolution and Function of MADS-Box Transcription Factors in Plants. Int. J. Mol. Sci..

[63] Molesini B., Dusi V., Pennisi F., Pandolfini T. (2020). How Hormones and MADS-Box Transcription Factors Are Involved in Controlling Fruit Set and Parthenocarpy in Tomato. Genes.

[64] Meng L., Zhang S., Chen B., Bai X., Li Y., Yang J., Wang W., Li C., Li Y., Li Z. (2021). The MADS-box transcription factor GlMADS1 regulates secondary metabolism in Ganoderma lucidum. Mycologia.

[65] Saavedra Núñez G., González-Villanueva E., Ramos P. (2023). Floral Development on Vitis vinifera Is Associated with MADS-Box Transcription Factors through the Transcriptional Regulation of VviZIP3. Plants.

[66] Abraham-Juárez M.J., Schrager-Lavelle A., Man J., Whipple C., Handakumbura P., Babbitt C., Bartlett M. (2020). Evolutionary Variation in MADS Box Dimerization Affects Floral Development and Protein Abundance in Maize. Plant Cell.

[67] Duan S.F., Yu J.C., Baldwin T.C., Yuan Y., Xiang G.S., Cui R., Zhao Y., Mo X.C., Lu Y.C., Liang Y.L. (2024). Genome-wide identification of a MADS-box transcription factor family and their expression during floral development in Coptis teeta wall. BMC Plant Biol..

[68] Qu Y., Kong W., Wang Q., Fu X. (2021). Genome-Wide Identification MIKC-Type MADS-Box Gene Family and Their Roles during Development of Floral Buds in Wheel Wingnut (*Cyclocarya paliurus*). Int. J. Mol. Sci..

[69] Smaczniak C., Immink R.G.H., Angenent G.C., Kaufmann K. (2012). Developmental and evolutionary diversity of plant MADS-domain factors: Insights from recent studies. Development.

[70] Ye L.X., Zhang J.X., Hou X.J., Qiu M.Q., Wang W.F., Zhang J.X., Hu C.G., Zhang J.Z. (2021). A MADS-Box Gene CiMADS43 Is Involved in Citrus Flowering and Leaf Development through Interaction with CiAGL9. Int. J. Mol. Sci..

[71] Ferrario S., Busscher J., Franken J., Gerats T., Vandenbussche M., Angenent G.C., Immink R.G. (2004). Ectopic expression of the petunia MADS box gene UNSHAVEN accelerates flowering and confers leaf-like characteristics to floral organs in a dominant-negative manner. Plant Cell.

[72] Tian Y., Dong Q., Ji Z., Chi F., Cong P., Zhou Z. (2015). Genome-wide identification and analysis of the MADS-box gene family in apple. Gene.

[73] Qian W., Liao B.-Y., Chang A.Y.-F., Zhang J. (2010). Maintenance of duplicate genes and their functional redundancy by reduced expression. Trends Genet..

[74] Meng D., Cao Y., Chen T., Abdullah M., Jin Q., Fan H., Lin Y., Cai Y. (2019). Evolution and functional divergence of MADS-box genes in Pyrus. Sci. Rep..

[75] Liu Y., Guan C., Chen Y., Shi Y., Long O., Lin H., Zhang K., Zhou M. (2024). Evolutionary analysis of MADS-box genes in buckwheat species and functional study of FdMADS28 in flavonoid metabolism. Plant Physiol. Biochem..

[76] Lucibelli F., Valoroso M.C., Theißen G., Nolden S., Mondragon-Palomino M., Aceto S. (2021). Extending the Toolkit for Beauty: Differential Co-Expression of DROOPING LEAF-Like and Class B MADS-Box Genes during Phalaenopsis Flower Development. Int. J. Mol. Sci..

[77] Eulgem T., Rushton P.J., Robatzek S., Somssich I.E. (2000). The WRKY superfamily of plant transcription factors. Trends Plant Sci..

[78] Wang Y., Zhou H., He Y., Shen X., Lin S., Huang L. (2023). MYB transcription factors and their roles in the male reproductive development of flowering plants. Plant Sci..

[79] Panchy N., Lehti-Shiu M., Shiu S.-H. (2016). Evolution of gene duplication in plants. Plant Physiol..

[80] Kumar G., Arya P., Gupta K., Randhawa V., Acharya V., Singh A.K. (2016). Comparative phylogenetic analysis and transcriptional profiling of MADS-box gene family identified DAM and FLC-like genes in apple (Malus × domestica). Sci. Rep..

[81] Jiménez S., Lawton-Rauh A.L., Reighard G.L., Abbott A.G., Bielenberg D.G. (2009). Phylogenetic analysis and molecular evolution of the dormancy associated MADS-box genes from peach. BMC Plant Biol..

[82] Cai X., Zeng X.L., Wang X.Q., Pan D., Zhang J., Li Z.Q., Yang J., Zhang Y.T., Zeng J., Zhang Q. (2025). Hormone metabolic profiling and transcriptome analysis reveal phytohormone crosstalk and the role of OfERF017 in the flowering and senescence of sweet osmanthus. Hortic. Plant J..

[83] Zhang Y.T., Yang J., Zeng X.L., Cai X., Li Z.Q., Zeng J., Zhang Q., Chen H.G., Zou J.J. (2025). Exploring miRNA-target modules regulating flower opening and senescence in Osmanthus fragrans through integrated transcriptome, miRNAome, and degradome analysis. Ind. Crops Prod..

