# Supplementary material for: Integrated Transcriptome and Metabolome Analyses Reveal the Roles of MADS-Box Genes in Regulating Flower Development and Metabolite Accumulation in Osmanthus fragran
Source: Curr Issues Mol Biol. 2025 Oct 3;47(10):819. doi: 10.3390/cimb47100819 (PMC12564812; doi:10.3390/cimb47100819)
Supplement: Supplementary file 1 [file cimb-47-00819-s001.zip › Supplemental figures.pdf]

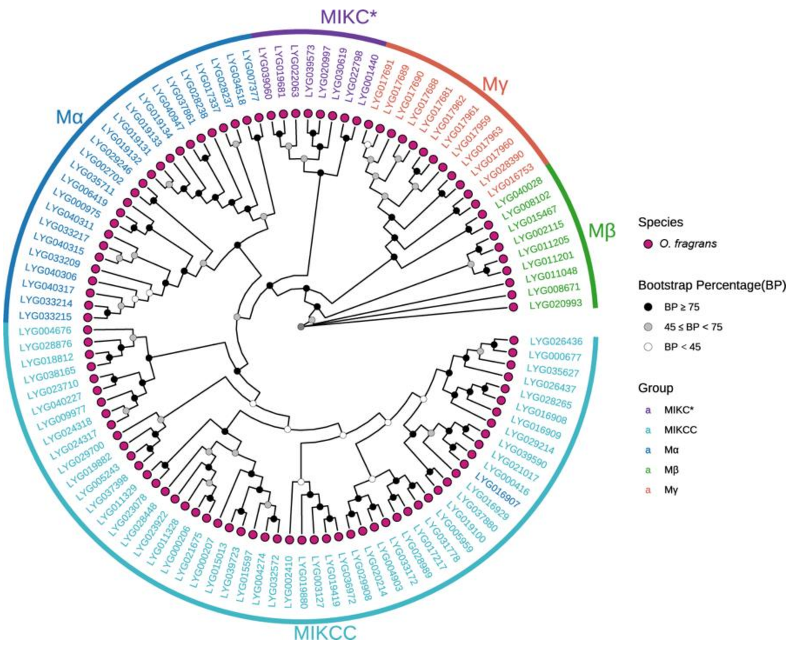

Supplemental Figure 1. The evolutionary examination of the 107 *OfMADS* protein sequences. These 107 OfMADS proteins were categorized into four distinct subgroups, labeled *MIKC*, MIKCC, Mα, Mβ* and *Mγ.* The evolutionary phylogeny was analyzed utilizing IQ-TREE software (2.0.3), employing the maximum likelihood (ML) method with bootstrap values derived from 1000 replicates. The phylogenetic tree was visualized through the use of the R package ggtree (3.10.0).

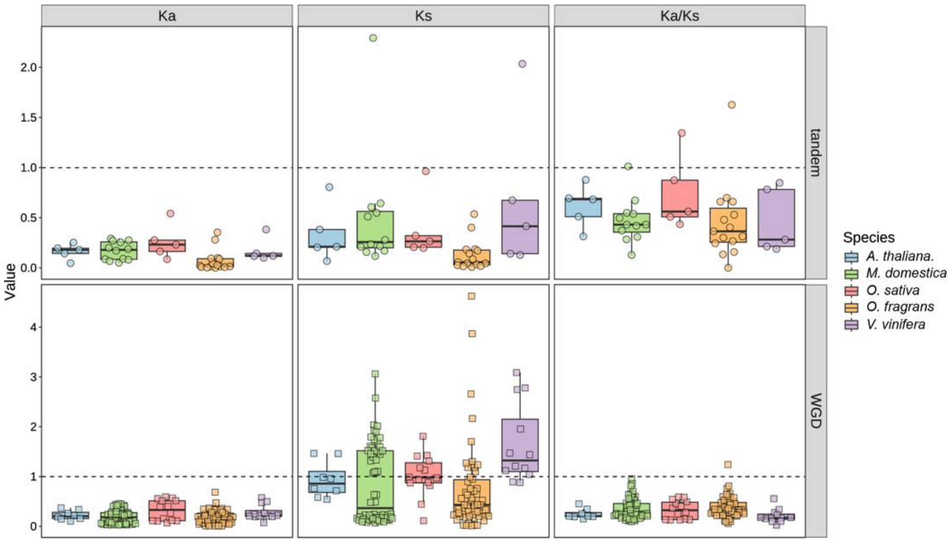

Supplemental Figure 2. Ka/Ks and Ka/Ks values of MADS duplicate gene pairs in *O. fragrans*, *O. sativa*, *M. domestica* and *V. vinifera* and *A. thaliana*.

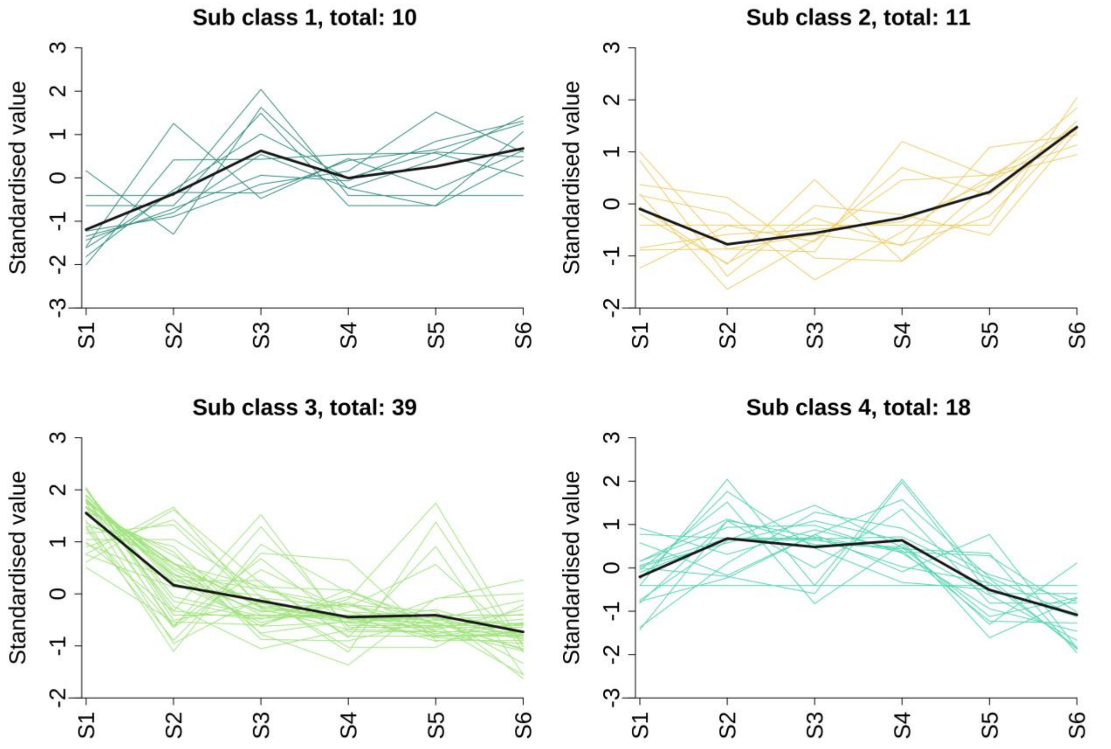

Supplemental Figure 3. Kmeans Analysis of Transcriptional Clustering Results Across Six Flowering Stages in *O. fragrans*. S1, S2, S3, S4, S5, and S6 correspond to the bolting stalk stage, early flowering stage, pre-flowering stage, full flowering stage, post-flowering stage, and shedding stage, respectively.

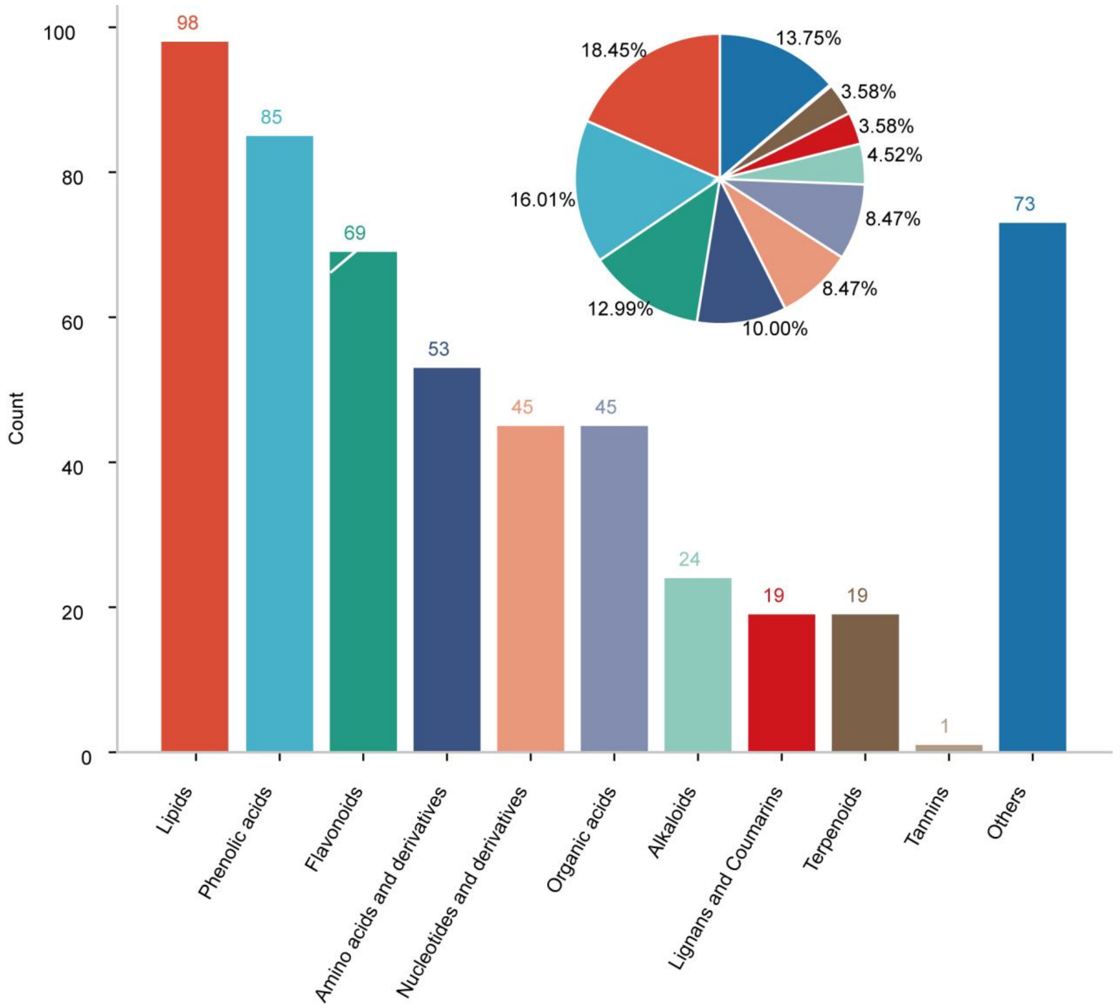

Supplemental Figure 4. Metabolite Classification Diagram Across Six Flowering Stages in *O. fragrans*.
